# Screening for and surveillance of premalignant conditions of the stomach

**DOI:** 10.1016/j.bpg.2025.101978

**Published:** 2025-01-15

**Authors:** Irina Luzko, Leticia Moreira, Jan Bornschein

**Affiliations:** a Department of Gastroenterology, Fundació de Recerca Clínic Barcelona-Institut d’Investigacions Biomèdiques August Pi i Sunyer (FRCB-IDIBAPS), CIBEREHD, Spain; b Facultat de Medicina, Universitat de Barcelona, Barcelona, 08036, Spain; c Medical Research Council Translational Immune Discovery Unit (MRC TIDU), Weatherall Institute of Molecular Medicine (WIMM), University of Oxford, Oxford, UK; d Translational Gastroenterology and Liver Unit, John Radcliffe Hospital, University of Oxford, Oxford, UK

**Keywords:** Gastric cancer, Surveillance, Screening, Early diagnosis

## Abstract

Strategies for population-based screening are thus far only implemented in high incidence countries such as South Korea or Japan with data showing a positive impact on gastric cancer mortality. Screening in most Western countries is deemed not cost-effective as these are mainly classified as low or intermediate risk regions. This is in part due to high costs for endoscopy as the diagnostic gold-standard. Blood testing for serum pepsinogens is implemented as a pre-screening tool in some Asian countries but can better highlight mucosal atrophy than the cancer itself. Endoscopic surveillance of patients with advanced preneoplastic conditions allows detection of early neoplastic lesions that can be treated endoscopically, resulting in better outcome, and is hence now also recommended by several European countries. However, there is no uniform approach, so screening and surveillance strategies need to take regional characteristics into account including gastric cancer incidence and cost for endoscopy among others.

## 1 Introduction

Gastric cancer (GC) is the fifth most common cancer worldwide and the second most frequent gastrointestinal tumor. In 2022, hotspots for GC were reported in Asia (691,791 cases), Europe (135,610 cases), and Latin America and the Caribbean (74,379 cases). The age-standardized incidence rate (ASR) per 100,000 was highest in Asia [11], followed by Latin America (8.5) and Europe (7.9) [1]. Within Europe, countries like Belarus, Russia, and Lithuania had higher ASR incidences (11.7–15.3), while Spain, Germany, and the UK had lower rates (3.6–6.8). Areas with ASR incidences below 10/100,000 are considered low-risk, 10–20 moderate, and above 20 high-risk [2].

Despite a decrease in incidence, GC remains the fifth leading cause of cancer mortality and incidence may rebound due to aging populations. In 2022, mortality rates were higher in Spain (12.8), Asia [10], and Latin America (8.7) [1]. Early GC is often asymptomatic, so diagnosis is often made in advanced stages leading to poor prognosis, with a 5-year survival rate below 7 % for metastatic cases, compared to 75 % for localized GC [3].

## 2 Premalignant lesions

As described by Pelayo Correa decades ago, atrophy and intestinal metaplasia are premalignant conditions in the carcinogenesis of intestinal type-gastric adenocarcinoma [4].

Gastric mucosal atrophy has been traditionally defined as ‘loss of proper gastric glands’, and two main types of atrophy can be recognized – metaplastic and non-metaplastic [5]. The mucosa may be replaced with fibrosis (non-metaplastic atrophy) or be replaced with intestinal or pseudo-pyloric mucosa (metaplastic).

Gastric intestinal metaplasia (GIM) is the replacement of proper gastric glands with mature intestinal cells as an adaptation to tissue injury; moreover, it is a precursor in the dysplasia–cancer sequence [6]. The most widely accepted classification of GIM distinguishes complete type metaplasia which resembles a small intestinal phenotype from the incomplete type which exhibits features of colonic epithelium and carries a higher risk of cancer development and the mixed type with both features, even in the same biopsy [7]. Prevalence of GIM has been reported from 3.4 % to 30 % in different series [8], Marques-Silva et al. reported a prevalence of 25 % (19–30 %) in a meta-analysis that included 107 studies performed in low and high-risk populations and more than 30,000 patients [9]. Up to date, no international guideline recommends screening for GIM per se in the general population [10].

Dysplasia has been described as an epithelium with cytological and architectural abnormalities, or as phenotypically neoplastic epithelium confined to glandular structures within the basement membrane [11]. Dysplasia can be categorized in low-grade dysplasia and high-grade dysplasia – equivalent to carcinoma in situ – depending on the severity of the cytological and architectural abnormalities. The term ‘indefinite for dysplasia’ is recommended to be used when it cannot be defined whether a lesion is neoplastic or non-neoplastic due to artefacts or inflammation in the sample, and the term ‘suspicion of invasive carcinoma’ for when it is hard to determine whether invasion into the lamina propria is present [12]. Indefinite for dysplasia should not be considered a minor finding. Risk factors such as larger size (>10 mm) and surface redness may be considered in patients with a diagnosis of ‘indefinite for dysplasia’ by endoscopic forceps biopsy, as up to 21 % of reveal underdiagnosed early gastric cancer [13].

## 3 Rationale for gastric screening

Approaches to tackle the challenges of both primary and secondary prevention of GC focus mainly on the *Helicobacter pylori* (*H. pylori*) infection, and the molecular changes that are driven by the infection-induced chronic inflammation [14]. While usual suspects for additional risk factors such as tobacco smoking, dietary factors and family history contribute to the overall individual risk [15–17], the sequence of preneoplastic conditions [4] generates opportunities for early detection strategies. First results indicate that this strategy reduces GC mortality [18], and long-term results are supposed to show an impact similar to the effects on oesophageal cancer mortality attributed to the surveillance of Barrett-metaplasia, a preneoplastic condition with features comparable to GIM [19]. However, it needs to be acknowledged that the main effect is reflected in reduced mortality rather than GC incidence rates, in comparison with colorectal cancer screening where endoscopic resection of precursor lesions reduce incidence by 52 % and mortality by 62 % [20]. Ablation or removal of atrophic mucosa or GIM is not feasible, but optimised surveillance regimens will increase the rate of early detection of both dysplastic lesions and intramucosal cancer, that can be treated curatively with minimally invasive measures.

Population-based screening for GC is only deemed reasonable in high-risk, or rather high incidence areas [21]. Problems are not only the risks associated with the invasive endoscopic procedure, but also the high costs for endoscopy in most countries. The latter would be overcome by the availability of a reliable and affordable non-invasive screening tool to identify individuals at high risk that would then benefit from further endoscopic work-up [22,23]. However, the tools currently available for gastric screening are limited.

## 4 Tools for gastric screening and established strategies

Upper GI endoscopy remains the gold standard for GC screening, outperforming upper GI radiology in sensitivity and clinical relevance. Its ability to obtain biopsies enables both diagnosis and individual risk stratification, making it more cost-effective than radiology [24]. Any analysis needs to consider the huge international variation regarding the costs for endoscopic tests, which also need to be factored into the assessment of national resources. Ascherman et al. presented a comprehensive analysis of data from countries representing five different continents [25]. They compared different screening scenarios with and without further endoscopic surveillance of high-risk patients and concluded that endoscopic screening can be cost-effective in high-incidence countries with low costs for screening (i.e. endoscopy) as well as in selected high-risk populations in low incidence countries. The definition of such high-risk criteria that allow the selection of a target population is a matter of debate and numerous research projects.

Thus far, no reliable, non-invasive test is available for GC detection. The only blood-based marker that has been extensively investigated is pepsinogen (PG) I as well as the ratio of pepsinogen I to II (PGr). PGI is exclusively produced in the chief cells of the gastric corpus and is hence reduced in case of glandular atrophy. PGII originates also from the prepyloric antrum, the cardia and the Brunner’s glands of the duodenum. It is increased in case of inflammatory conditions such as *H. pylori* infection [26]. The diagnostic quality of PG serology as a marker obviously depends on the cut-offs chosen [27]. While PG testing appears to be an appropriate tool for non-invasive diagnosis of atrophic changes of the gastric mucosa [28], its properties as a GC screening marker are not sufficient. Recent meta-analyses attest pooled sensitivities of 57–59 % and specificities of 73–87 % [28,29]. Often, PG testing is combined with assessment of both *H. pylori* serology as well as serum gastrin 17 (G17) levels, with the latter representing a marker of antral gastric atrophy, but the diagnostic yield remains similar to PG testing alone [30,31]. Hence, PG testing is mostly used now as pre-screening tool to identify those that require further endoscopic assessment as outlined below [32].

Thus far, no superior diagnostic marker has been identified. Trefoil factor 3 (TFF3) in the serum has been discussed for a while but too many confounders impact sensitivity and specificity in particular [33,34].

## 5 Strategies for gastric cancer screening

Screening should be universal to achieve the highest impact on public health, but factors like national resources, regional incidence of the disease, and the cost-effectiveness of tools affect feasibility. Population-based screening must be cost-effective, address social inequalities, ensure clear communication, and require proper governance and quality assurance. Implementation of the strategies often depends on cost-effectiveness [35]. The studies in this field are often unidimensional, targeting the cancer as hard endpoint (i.e. incidence and mortality). Furthermore, the landscape of treatment options for malignant diseases has rapidly developed over the last decades leading to better prognosis for many entities, but also more complex and expensive treatment regimens. Since the outcome remains stage-dependent for most cancers, a recent meta-analysis of screening efforts led by IARC suggests considering the detection rate of late-stage cancers as alternative endpoint for some entities [36]. These issues have been recognized for the stomach, where screening does not only target detection of cancer, but also preneoplastic conditions [37].

Thus far, population-based screening for gastric cancer has only been implemented in East Asia, primarily in Japan and South Korea. A recent analysis compared the impact of both national screening programs against data from a synthetic control cohort including data from countries without structured screening programs [18]. The RR for GC mortality in the screening population was 0.83 (95 % CI: 0.71–0.96) for South Korea and 0.97 (95 % CI: 0.88–1.07) for Japan. There was also a good effect on mortality due to other upper gastrointestinal disease (RR 0.72, 95 % CI: 0.57–0.90) in South Korea, but not Japan (RR 0.93, 95 % CI: 0.68–1.28). Potential factors causing the discrepancy between both countries are the use of different screening modalities (mainly radiology tests in Japan) and that adherence to the screening guideline was mandatory only in South Korea [18]. Overall, the effect remains lower than expected from a previous meta-analysis [38].

There are no real-life data from such approaches in low-risk countries, so most strategies are inferred from cost-effectiveness modelling of low-risk scenarios [39,40]. Such studies suggest that targeted screening might be more feasible in low or intermediate risk areas, referring to a pre-selection of the target population based on specific risk factors -such as age, sex, smoking habits, persistent *H. pylori* infection, pernicious anaemia and family history for GC [41,42]. Ethnicity can serve a selection marker in conjunction with age, but there are doubts if this effect is pertained in second or third generation immigrant families [40,43]. Family history itself has been confirmed as relevant risk factor with a recent meta-analysis with 21 included studies confirming a three-fold risk increase for first degree relatives of GC patients (OR 2.92, 95 % CI: 2.402–3.552) [17]. A retrospective cohort study from the US demonstrated that the likelihood for missed or interval cancers in a gastric surveillance cohort was higher if additional risk factors were present at the time of the index endoscopy. In this study, 92.6 % and 76.9 % of the 91 patients with interval GC, carried at least one or two additional risk factors, respectively [44].

As mentioned above, some strategies rely on pre-selection of individuals at risk by serology. In Asia, the combination of serum PG assessment and the serological *H. pylori* status is used to stratify the patients into risk cohorts. Most common is the so-called ABC(D) method as described by Watabe et al. 2005 [45]. In this Japanese study, 9293 subjects were stratified in four groups with different attributable GC risk in a 4.7 years follow-up: *A* – normal PG/negative H. pylori, *B* – normal PG/positive *H. pylori* (HR 1.1 [95 % CI: 0.4–3.4], *C* – pathological PG/positive H. pylori (HR 6.0 [95 % CI: 2.4–14.5]), and *D* – pathological PG/negative H. pylori (HR 8.2 [95%CI: 3.2–21.5]). A meta-analysis reported a risk increase by 6-60-fold in subjects with both positive PG-test and *H. pylori* serology [46]. In Asian populations, still with no *H. pylori* status included, serological preselection followed by endoscopy in positive cases has shown to have the potential to reduce GC mortality by 76 % [47].

Regarding alternative strategies to improve cost-effectiveness of GC screening in low or intermediate risk countries, the United European Gastroenterology (UEG) federation published in 2022 a position paper suggesting combining upper GI screening with established colorectal cancer screening pathways, also in agreement with the recent European Maastricht/Florence consensus [41,48]. Pilot studies from France, Germany and Slovenia have demonstrated that this approach is feasible, applying either direct upper GI endoscopy for FIT positive patients or serology as pre-screening tool in those undergoing colorectal cancer screening, then followed by gastroscopy of positive subjects [49–51]. Data from the US showed that the former can increase the detection of malignant GI disease by 25 % [52].

Until that benefits of either population-based or targeted screening have been fully confirmed and such strategies established, opportunistic screening remains the most common approach in most countries. This includes careful assessment of the stomach, protocol-based biopsy sampling and appropriate photo-documentation [53]. Despite clear guidance, both prospective and retrospective studies have shown that adherence to sampling protocols is rather low, so there is a clear need to emphasize the importance of this topic and facilitate better training [54, 55].

Virtual chromoendoscopy (VCE) can improve the diagnostic yield of targeted biopsies in this setting, but studies do not fully support yet abandoning random biopsies [56–61]. The application of established, validated classification system is beneficial [62–64], there is increasing evidence that dedicated training of upper endoscopy and systematic mucosal inspection has a positive impact on lesion detection rates [65–68]. Despite the efforts to implement new strategies in clinical practice, there is still a high rate of missed cancers (6–9%) [69,70]. There is hope that artificial intelligence (AI) systems will contribute to the reduction of missed GC [71]. Recent meta-analyses attest the application of AI systems a sensitivity of 87–88 % and a specificity of 88–89 % for GC detection [72,73]. Currently, there remains some related issues such as the training of AI systems is still vastly based on images rather than videos, and the algorithms also depend on expert input as ‘gold-standard’ which shows significant interobserver variability. Nevertheless, evidence suggest that the introduction of AI in certain settings can improve the cost-effectiveness of upper GI screening even in low or intermediate risk countries in Europe [74].

## 6 Surveillance

Patients with premalignant gastric lesions are at risk to develop GC, which depends directly on the extent and severity of atrophy and GIM. The annual risk of progression to GC has been described by de Vries et al. as ranging from 0.6 % to 6 % for dysplasia, 0.25 % for GIM, and 0.1 % for gastric atrophy [75].

### 6.1 Risk stratification

#### 6.1.1 Histological risk assessment

*The Sydney Consensus*, proposed in 1991 and revised in 1994, defined the standardization of gastritis based on aetiology, morphology, and topography of the inflammation with separate examination of the antrum and corpus of the stomach [76]. The updated version suggested taking biopsies from both the lesser and the greater curvature of the antrum and corpus, and from the incisura, assessing five key features in histological reports: *H. pylori* presence, chronic inflammation, activity, atrophy, and GIM, each graded as not present, mild, moderate, or severe. However, this histological information lacks utility for predicting GC risk [77].

In 2005, Rugge et al. proposed the *Operative Link on Gastritis Assessment* (OLGA) system for a histology-based GC risk stratification (Table 1). OLGA stratifies gastritis in stages by combining the degree of atrophy with its anatomical distribution on a scale that reflects an increasing risk of GC from lowest (OLGA stage 0) to the highest risk (OLGA state IV) [77]. Subsequently, the OLGIM score was proposed based on the degree and extension of GIM, replacing gastric atrophy (Table 1) [78]. Both systems can only be applied when a complete set of biopsy samples is available as determined by the mapping protocol including corpus, antrum and incisura [79].

The OLGIM score shows a higher intra- and interobserver agreement than OLGA and should be preferred when the aim is staging of mucosal changes [78,80]. The extent of GIM is correlated to the proportion of incomplete GIM. In the same way, the prevalence of incomplete GIM significantly increase with higher OLGA/OLGIM stages (stages III/IV) [81,82].

Rugge et al. reported data on 7436 patients that were followed-up for a median of 6.6 years. Only 2.3 % of those enrolled showed advanced high-risk stages of atrophy (OLGA III/IV) with a HR for GC of 712 (95 % CI: 92.5–5484.5) for stage III and a HR of 1450 (95 % CI: 166.7–12626.0) for stage IV [83]. Meta-analyses confirm the applicability of both staging systems, although the calculated attributable risk of progression is lower than initially expected, which might be due to a considerable impact of study heterogeneity and the fact that diffuse type cancers were included in many studies (OR for GC for OLGA III/IV: 2.64, 95%CI; 1.84–3.79; OLGIM III/IV: 3.99, 95%CI: 3.05–5.21) [84,85].

#### 6.1.2 Endoscopic risk assessment

The *Kimura Takemoto* classification is a visual scale frequently used in Eastern countries, based on the endoscopic recognition of the atrophic border by distinguishing differences in colour and level of the gastric mucosa. Atrophy is characterized by a pale yellowish colour and increased visibility of vessels in the mucosa [86]. The Kimura Takemoto system categorizes atrophy into two primary types reflecting the progression of atrophy from the distal to the proximal part of the stomach: the ‘closed’ types where gastric atrophy is mainly confined to the antrum and does not extend into the gastric corpus (C0, C-1, C-2, and C-3), and the ‘open’ types, where gastric atrophy has extended into the corpus, moving towards the greater curvature of the stomach, O-1 represents atrophy that has just begun to spread into the corpus, O-2 indicates moderate extension, and O-3 indicates that atrophy has spread extensively [86].

The *Endoscopic Grading of Gastric Intestinal Metaplasia* (EGGIM) system is based on the endoscopic grading of the severity of GIM using Narrow band imaging (NBI) and has shown excellent diagnostic performance according to two recent meta-analyses [84,87]. Using NBI, GIM is mainly characterized by a regular tubulo-villous pit pattern and the presence of the ‘Light Blue Crest’ sign [88]. EGGIM evaluates five different areas (lesser and grater curvature of the antrum and the corpus plus the incisura) adding scores of 0 (no GIM), 1 (≤30 % GIM) or 2 (>30 % GIM) for each area. A higher score indicates a higher risk of developing GIM [89]. The EGGIM score is highly consistent with OLGIM, and patients with EGGIM score of 5–10 are at a higher risk for early GC [90].

### 6.2 Strategies

In addition to *H. pylori* eradication, the main strategy to reduce the risk of GC in patients with premalignant lesions is endoscopic surveillance and resection of high-risk lesions [91]. Thus, it is essential to identify those at higher risk of progression to provide appropriate surveillance and to offer earlier and less invasive treatment [77]. Most of current guidelines agree in two main recommendations: implementation of histology-based risk-stratification to direct surveillance and mandating *H. pylori* eradication when present. Beside those, guidelines may vary mainly depending on the incidence of GC in each region or country. The guidelines covered throughout the present manuscript are summarized in Table 2.

#### 6.2.1 Surveillance for gastric mucosal atrophy

In terms of gastric mucosal atrophy surveillance, several guidelines such as the German, Italian, American and the European *Management of epithelial precancerous conditions and lesions in the stomach* (MAPS II), agree that there is no evidence to recommend surveillance for people with mild to moderate atrophy restricted to the antrum [80,91–94]. However, patients with advanced stages of atrophy (severe atrophic changes in both the antrum and corpus, OLGA III/IV) are recommended to undergo high-quality endoscopy every two to three years [80,92,93]. Those with GC family history may benefit from more frequent follow-up, such as every 1–2 years after diagnosis [80].

Similarly, the British Society of Gastroenterology recommends a 3-year interval for endoscopic surveillance in people with extensive gastric atrophy, regardless of the presence of risk factors. For cases limited to the antrum, the same interval is recommended if there is a family history of GC or persistent *H. pylori* infection [95].

Otherwise, the Spanish national guideline does not recommend endoscopic surveillance of patients with atrophy without metaplasia, independently of the extension [91].

#### 6.2.2 Surveillance of patients with GIM

In this setting, according to MAPS II, the German and the Italian guidelines, patients with moderated to marked features of GIM OLGIM III/IV should be followed up with a high-quality endoscopy every 3 years. For patients with GIM at a single location, endoscopic surveillance is only recommended if there are additional risk factors such as persistent *H. pylori* infection, incomplete GIM or autoimmune gastritis [80].

According to British and American recommendations, earlier endoscopic surveillance is advised for focal GIM if additional risk factors are present. The British guidelines include a strong family history of GC or persistent *H. pylori* infection as such risk factors, the American guidelines list a positive family history, incomplete GIM, high-risk ethnicity, or immigration from high-incidence regions. They also recommend endoscopic surveillance for patients with extensive GIM(92,95). In those for whom endoscopic surveillance is indicated, the recommended interval is 3 years [95].

In higher risk areas such as Chile, annual endoscopic surveillance is recommended including biopsies in patients with incomplete GIM or OLGIM III/IV. For patients with OLGIM I-II or mild GIM with no others risk factors endoscopic surveillance should be done every three years [96].

Conversely, the Spanish guidelines do not recommend endoscopic surveillance for patients with focal GIM (OLGIM I-II), nor for extensive GIM (OLGIM III-IV) in patients without incomplete GIM or first-degree family history of GC. For patients with incomplete GIM or a firstdegree risk family history of GC, endoscopic surveillance is recommended every 3 years [91]. If the GIM diagnosis was made with low-quality endoscopy, it is advised to repeat the endoscopy (with high quality and OLGIM scoring in the report) within 6–12 months [91].

#### 6.2.3 Surveillance for dysplasia

When dysplasia is found in a biopsy from an endoscopically visible lesion is recommended a high definition chromoendoscopic reassessment at a reference centre. If the lesion is confirmed, this should be resected (endoscopically) to ensure an accurate histological diagnosis [80]. After endoscopic treatment, annual endoscopic surveillance is suggested for 2 years, followed by further monitoring based on the baseline risk after the endoscopic resection of a gastric adenoma with low-grade dysplasia [80,91].

For patients with dysplasia identified in a ‘random’ biopsy, i.e. without an identifiable lesion on endoscopy, an immediate high-quality endoscopic re-evaluation is advised [80,95]. If no lesion is detected during this procedure, biopsies for gastritis staging (if not previously performed) and a follow-up endoscopy are suggested within 6 months or 12 months for high-grade and low-grade dysplasia, respectively [80,91, 95].

**Fig. 1:**
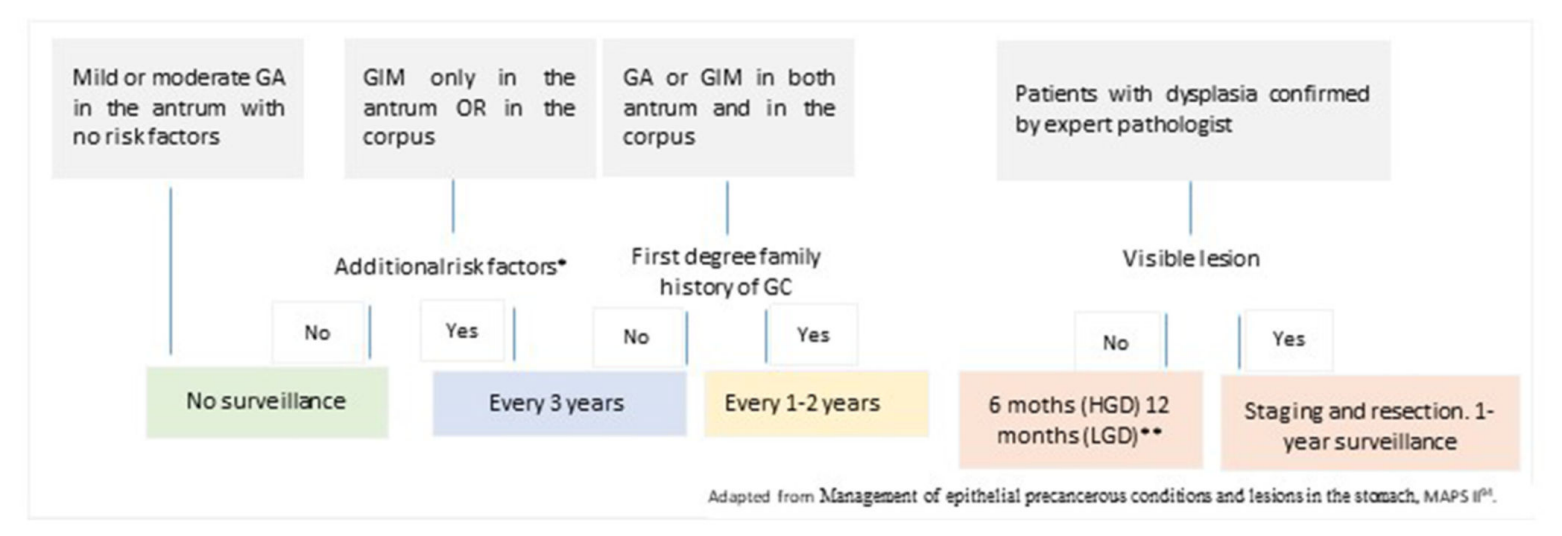
Proposed endoscopic surveillance for patients with pre neoplastic conditions.

Based on all the previously mentioned recommendations, and mainly on MAPS II, an endoscopic surveillance strategy is proposed for patients from low-to-intermediate risk areas (Fig. 1).

### 6.3 Pitfalls in surveillance of patients with premalignant lesions

Some issues have not yet conclusively resolved regarding the surveillance of premalignant lesions. One of them is to determine an appropriate age threshold at which not to start or to stop surveillance. Endoscopic surveillance of premalignant lesions is justified for patients who are likely to benefit from early detection, meaning they are candidates for curative or prognosis-altering treatment. European guidelines advise against surveillance in individuals over 75–80 years old and/or with a life expectancy of less than 10 years and poor general health status. In those it is considered unlikely to alter life expectancy significantly — one of the main objectives of the screening [91,97].

Regarding to specific populations at risk, some authors consider that autoimmune gastritis confers higher risk for GC development [98,99]. Although molecular pathways involved are not clear and there is heterogeneity between published cohorts, the MAPS II consensus recommends a follow-up endoscopy at 3- to 5-year intervals in patients with autoimmune gastritis [80]. Another unfilled gap in this setting is the absence of specific guidance regarding the management of GIM in special situations such as hereditary syndromes, a post-surgical stomach or after endoscopic resection of neoplasia [10].

Furthermore, there is insufficient evidence to make a uniform statement regarding the cost-effectiveness of surveillance in western countries. A study performed in United States among patients diagnosed with GIM and dysplasia suggested that endoscopic resection with annual surveillance reduced lifetime cancer risk by 90 % and cost $39,800 per quality-adjusted-life-year [100]. In the absence of clinical trials on this topic, Lansdor-Vogelaar et al. suggest that cost-effectiveness of upper endoscopy for surveillance of patients with GIM is uncertain but may be effective in high-risk groups in Western countries [101]. A systematic review of cost-effectiveness studies was performed by Areia et al. reporting also conflicting results between included articles on the follow up of pre-malignant lesions [102]. To continue evaluating this point, more studies and stricter implementation of published guidelines are needed.

## 7 Conclusions

Early-stage diagnosis of GC is one of the key factors in improving survival rates. It has been suggested by several guidelines that in addition to eradicating *H. pylori* in infected patients, detecting premalignant lesions offers an opportunity to influence the carcinogenesis sequence, offering endoscopic treatment and ultimately improving cancer survival.

However, there are no reliable non-invasive markers for GC screening. Thus, endoscopic examination remains the gold standard for diagnosing GC. As mentioned above, identifying patients with a higher risk of premalignant conditions allows their inclusion in endoscopic surveillance with different interval regimens adapted to individual risk, which are now recommended by several European countries.

Opportunistic screening seems to remain the most common approach, although the adherence to sampling protocols is rather low. Emphasize the importance of better training and implementation of the targeted screening—such as selecting individuals with additional risk factors or combining it with established screening programs like colorectal cancer screening— may improve the cost-effectiveness and feasibility of screening approaches in low- or intermediate-risk areas.

Prospective, multicenter studies are needed to establish the best strategy for GC screening and surveillance, addressing who, how and when to screen.

## Figures and Tables

**Table 1:** OLGA/OLGIM staging system [78].

		Corpus			
**Antrum** ^a^	Atrophy/ GIM score	Absent (score 0)	Mild (score 1)	Moderate (score 2)	Severe (score 3)
	Absent (score 0)	Stage 0	Stage I	Stage II	Stage II
	Mild (score 1)	Stage I	Stage I	Stage II	Stage III
	Moderate (score 2)	Stage II	Stage II	Stage III	Stage IV
	Severe (score 3)	Stage III	Stage III	Stage IV	Stage IV

GIM, gastrointestinal metaplasia; OLGA, Operative link on gastritis assessment; OLGIM, Operative link on gastric intestinal metaplasia assessment.

a Including incisura angularis.

**Table 2:** Current guidelines and consensus documents with recommendations on endoscopic surveillance intervals for gastric premalignant lesions.

Guidelines/ Consensus	Gastric Mucosal Atrophy	Gastrointestinal Metaplasia	Dysplasia (1)
**MAPS II** [80] **(2019)**	Limited, OLGA I-II: No	Limited ^(only if2,3 or4)^: 3 years	LGD: 1y
	Extensive, OLGA III-IV: 3 years (or 1 - 2y ^if2^)	Extensive, OLGIM III-V: 3 years (or 1 - 2y^if2^)	HGD: 6 mo.
**Spain** [91] **(2021)**	No	OLGIM I - II: No	LGD: 1y
		OLGIM III-IV ^if2 or4:^ 3 y	HGD: 6 mo.
**UK** [95] **(2019)**	Limited, OLGA I-II ^if2 or4:^ 3y	Limited, OLGIM I-II ^if2 or4:^ 3y	LGD: 1y
	Extensive, OLGA III- IV: 3y	Extensive, OLGIM III-IV: 3y	HGD: 6 mo.
**Italian** [93] **(2019)**	Limited, OLGA I-II: No	Limited, OLGIM I-II with no additional risk factors: No	NA
	Extensive, OLGA III- IV: 3y	Extensive, OLGIM III-IV: 3y	
**USA** [92] **(2021)**	Limited, OLGA I-II: No	Limited, OLGIM I-II ^if2,4 or5^: 3 to 5y	NA
	Extensive, OLGA III- IV: 3 to 5y	Extensive, OLGIM III-IV: 3 to 5y	
**Chile** [96] **(2014)**	OLGA I-II: 3y	OLGA I-II: 3 y	LGD: 1y
	OLGA III-IV: 1y	OLGA III-IV: 1y	HGD: 6 mo.
**China** [103] **(2022)**	Limited: No	OLGIM II: 5 y	NA
	Extensive: 1 to 3 y	OLGIM III-IV: 2 y Incomplete GIM: 3y	
**Germany** [94] **(2024)**	OLGA III-IV: 2–3 y	OLGIM III-IV: 2-3y	NA

1 Dysplasia with non-visible lesion.

2 Family history of gastric cancer.

3 *Helicobacter pylori* persistent infection.

4 Incomplete gastrointestinal metaplasia.

5 Immigrants from high incidence region, ethnic/racial minorities.

* Limited: limited to the antrum; Extensive: extensive to the corpus. Management of epithelial precancerous conditions and lesions in the stomach, MAPS II; United Kingdom, UK; United States of America; USA; Operative Link on Gastritis Assessment, OLGA; Operative link on gastric intestinal metaplasia assessment, OLGIM; Not addressed, NA; Gastric Cancer, GC; Gastrointestinal metaplasia, GIM; Low grade dysplasia, LGD; High grade dysplasia, HGD; years, y; months, mo.

## References

[1] World Health Organization IA for R on C (2022). Global cancer observatory. Cancer Today.

[2] Fock KM, Ang TL (2010). Epidemiology of *Helicobacter pylori* infection and gastric cancer in Asia. J Gastroenterol Hepatol.

[3] National Cancer Institute (2023). Stomach cancer survival rates.

[4] Correa P (1992). Human gastric carcinogenesis: a multistep and multifactorial process—first American cancer society award lecture on cancer epidemiology and prevention. Cancer Res.

[5] Rugge M, Correa P, Dixon MF, Fiocca R, Hattori T, Lechago J (2002). Gastric mucosal atrophy: interobserver consistency using new criteria for classification and grading. Aliment Pharmacol Ther.

[6] Giroux V, Rustgi AK (2017). Metaplasia: tissue injury adaptation and a precursor to the dysplasia–cancer sequence. Nat Rev Cancer.

[7] Shah SC, Gawron AJ, Mustafa RA, Piazuelo MB (2020). Histologic subtyping of gastric intestinal metaplasia: overview and considerations for clinical practice. Gastroenterology.

[8] Altayar O, Davitkov P, Shah SC, Gawron AJ, Morgan DR, Turner K (2020). AGA technical review on gastric intestinal metaplasia—epidemiology and risk factors. Gastroenterology.

[9] Marques-Silva L, Areia M, Elvas L, Dinis-Ribeiro M (2014). Prevalence of gastric precancerous conditions. Eur J Gastroenterol Hepatol.

[10] Dinis-Ribeiro M, Shah S, El-Serag H, Banks M, Uedo N, Tajiri H (2024). The road to a world-unified approach to the management of patients with gastric intestinal metaplasia: a review of current guidelines. Gut.

[11] Rugge M, Correa P, Dixon MF, Hattori T, Leandro G, Lewin K (2000). Gastric dysplasia. Am J Surg Pathol.

[12] Schlemper RJ (2000). The Vienna classification of gastrointestinal epithelial neoplasia. Gut.

[13] Goo JJ, Choi CW, Kang DH, Kim HW, Park SB, Cho M (2015). Risk factors associated with diagnostic discrepancy of gastric indefinite neoplasia: Who need en bloc resection?. Surg Endosc.

[14] Bornschein J, Bird-Lieberman EL, Malfertheiner P (2019). The rationale and efficacy of primary and secondary prevention in adenocarcinomas of the upper gastrointestinal tract. Dig Dis.

[15] Rota M, Possenti I, Valsassina V, Santucci C, Bagnardi V, Corrao G (2024). Dose–response association between cigarette smoking and gastric cancer risk: a systematic review and meta-analysis. Gastric Cancer.

[16] Liu SJ, Huang P Di, Xu JM, Li Q, Xie JH, Wu WZ (2022). Diet and gastric cancer risk: an umbrella review of systematic reviews and meta-analyses of prospective cohort studies. J Cancer Res Clin Oncol.

[17] Ligato I, Dottori L, Sbarigia C, Dilaghi E, Annibale B, Lahner E (2024). Systematic review and meta-analysis: risk of gastric cancer in patients with first-degree relatives with gastric cancer. Aliment Pharmacol Ther.

[18] Sun D, Mülder DT, Li Y, Nieboer D, Park JY, Suh M (2024). The effect of nationwide organized cancer screening programs on gastric cancer mortality: a synthetic control study. Gastroenterology.

[19] Ding YE, Li Y, He XK, Sun LM (2018). Impact of Barrett’s esophagus surveillance on the prognosis of esophageal adenocarcinoma: a meta-analysis. J Dig Dis.

[20] Zhang J, Chen G, Li Z, Zhang P, Li X, Gan D (2020). Colonoscopic screening is associated with reduced Colorectal Cancer incidence and mortality: a systematic review and meta-analysis. J Cancer.

[21] Lansdorp-Vogelaar I, Meester RGS, Laszkowska M, Escudero FA, Ward ZJ, Yeh JM (2021). Cost-effectiveness of prevention and early detection of gastric cancer in Western countries. Best Pract Res Clin Gastroenterol.

[22] Chan FKL, Wong MCS, Chan AT, East JE, Chiu HM, Makharia GK (2023). Joint Asian Pacific Association of Gastroenterology (APAGE)-Asian Pacific Society of Digestive Endoscopy (APSDE) clinical practice guidelines on the use of non-invasive biomarkers for diagnosis of colorectal neoplasia. Gut.

[23] Monahan KJ, Davies MM, Abulafi M, Banerjea A, Nicholson BD, Arasaradnam R (2022). Faecal immunochemical testing (FIT) in patients with signs or symptoms of suspected colorectal cancer (CRC): a joint guideline from the Association of Coloproctology of Great Britain and Ireland (ACPGBI) and the British Society of Gastroenterology (BSG). Gut.

[24] Kowada A (2019). Cost-effectiveness of Helicobacter pylori test and eradication versus upper gastrointestinal series versus endoscopy for gastric cancer mortality and outcomes in high prevalence countries. Scand J Gastroenterol.

[25] Ascherman B, Oh A, Hur C (2021). International cost-effectiveness analysis evaluating endoscopic screening for gastric cancer for populations with low and high risk. Gastric Cancer.

[26] di Mario F, Cavallaro LG (2008). Non-invasive tests in gastric diseases. Dig Liver Dis.

[27] Lee SY, Ahn YS, Moon HW (2024). Comparison between the GastroPanel test and the serum pepsinogen assay interpreted with the ABC method—a prospective study. Helicobacter.

[28] Bang CS, Lee JJ, Baik GH (2019). Prediction of chronic atrophic gastritis and gastric neoplasms by serum pepsinogen assay: a systematic review and meta-analysis of diagnostic test accuracy. J Clin Med.

[29] Faria L, Silva JC, Rodríguez-Carrasco M, Pimentel-Nunes P, Dinis-Ribeiro M, Libânio D (2022). Gastric cancer screening: a systematic review and meta-analysis. Scand J Gastroenterol.

[30] Gašenko E, Bogdanova I, Sjomina O, Aleksandraviča I, Kiršners A, Ancāns G (2023). Assessing the utility of pepsinogens and gastrin-17 in gastric cancer detection. Eur J Cancer Prev.

[31] Syrjänen K (2022). Accuracy of serum biomarker panel (GastroPanel®) in the diagnosis of atrophic gastritis of the corpus. Systematic review and meta-analysis. Anticancer Res.

[32] Sánchez-López JY, Díaz-Herrera LC, Rizo-de la Torre LDC, Pepsinogen I (2024). pepsinogen II. gastrin-17, and Helicobacter pylori serological biomarkers in the diagnosis of precursor lesions of gastric cancer. Arch Med Sci.

[33] Xu Y, Miremadi A, Link A, Malfertheiner P, Fitzgerald RC, Bornschein J (2019). Feasibility of combined screening for upper gastrointestinal adenocarcinoma risk by serology and Cytosponge testing: the SUGAR study. J Clin Pathol.

[34] Kaise M, Miwa J, Fujimoto A, Tashiro J, Tagami D, Sano H (2013). Influence of Helicobacter pylori status and eradication on the serum levels of trefoil factors and pepsinogen test: serum trefoil factor 3 is a stable biomarker. Gastric Cancer.

[35] Stefan Lönnberg, Mario Šekerija, Nea Malila, Tytti Sarkeala, Marcis Leja OM, Marco Zappa, Eveline Heijnsdijk, Sirpa Heinävaara, de Koning Aanttila Harry (2017). European guide on quality improvement in comprehensive cancer control.

[36] Feng X, Zahed H, Onwuka J, Callister MEJ, Johansson M, Etzioni R (2024). Cancer stage compared with mortality as end points in randomized clinical trials of cancer screening: a systematic review and meta-analysis. JAMA.

[37] Sui Z, Chen J, Li P, Shao L, Ye J, Lu X (2020). Risk for gastric cancer in patients with gastric atrophy: a systematic review and meta-analysis. Transl Cancer Res.

[38] Zhang X, Li M, Chen S, Hu J, Guo Q, Liu R (2018). Endoscopic screening in asian countries is associated with reduced gastric cancer mortality: a meta-analysis and systematic review. Gastroenterology.

[39] Areia M, Spaander MCW, Kuipers EJ, Dinis-Ribeiro M (2018). Endoscopic screening for gastric cancer: a cost-utility analysis for countries with an intermediate gastric cancer risk. United European Gastroenterol J.

[40] Shah SC, Canakis A, Peek RM, Saumoy M (2020). Endoscopy for gastric cancer screening is cost effective for asian Americans in the United States. Clin Gastroenterol Hepatol.

[41] Malfertheiner P, Megraud F, Rokkas T, Gisbert JP, Liou JM, Schulz C (2022). Management of Helicobacter pylori infection: the Maastricht VI/Florence consensus report. Gut.

[42] Nakamura K, Kakugawa Y, Sekiguchi M, Tsuruki ES, Matsumoto M, Hisada I (2024). Chronological trend of opportunistic endoscopic screening for gastric cancer and atrophic gastritis. Asian Pac J Cancer Prev APJCP.

[43] Saumoy M, Schneider Y, Shen N, Kahaleh M, Sharaiha RZ, Shah SC (2018). Cost effectiveness of gastric cancer screening according to race and ethnicity. Gastroenterology.

[44] Tan MC, Mallepally N, Nguyen TH, Hammad T, Kim DK, Othman MO (2023). Missed opportunities for screening or surveillance among patients with newly diagnosed non-cardia gastric adenocarcinoma. Dig Dis Sci.

[45] Watabe H, Mitsushima T, Yamaji Y, Okamoto M, Wada R, Kokubo T (2005). Predicting the development of gastric cancer from combining Helicobacter pylori antibodies and serum pepsinogen status: a prospective endoscopic cohort study. Gut.

[46] Terasawa T, Nishida H, Kato K, Miyashiro I, Yoshikawa T, Takaku R (2014). Prediction of gastric cancer development by serum pepsinogen test and Helicobacter pylori seropositivity in Eastern Asians: a systematic review and meta-analysis. PLoS One.

[47] Yoshihara M, Hiyama T, Yoshida S, Ito M, Tanaka S, Watanabe Y (2007). Reduction in gastric cancer mortality by screening based on serum pepsinogen concentration: a case-control study. Scand J Gastroenterol.

[48] Burra P, Bretthauer M, Buti Ferret M, Dugic A, Fracasso P, Leja M (2022). Digestive cancer screening across Europe. United European Gastroenterol J.

[49] Planade O, Dessomme B, Chapelle N, Verdier M, Duchalais E, Queneherve L (2021). Systematic upper endoscopy concomitant with colonoscopy performed within the colorectal cancer screening program: impact on the patients’ management. Clin Res Hepatol Gastroenterol.

[50] Selgrad M, Bornschein J, Kandulski A, Weigt J, Roessner A, Wex T (2018). Combined gastric and colorectal cancer screening-A new strategy. Int J Mol Sci.

[51] Tepes B, Seruga M, Vujasinovic M, Urlep D, Ljepovic L, Brglez JN (2017). Premalignant gastric lesions in patients included in National colorectal cancer screening. Radiol Oncol.

[52] Shah A, Eqbal A, Moy N, Koloski N, Messmann H, Kendall BJ (2023). Upper GI endoscopy in subjects with positive fecal occult blood test undergoing colonoscopy: systematic review and meta-analysis. Gastrointest Endosc.

[53] Rugge M, Genta RM, Malfertheiner P, Dinis-Ribeiro M, El-Serag H, Graham DY (2024). RE.GA.IN.: the real-world gastritis initiative-updating the updates. Gut.

[54] Lash JG, Genta RM (2013). Adherence to the Sydney System guidelines increases the detection of Helicobacter gastritis and intestinal metaplasia in 400 738 sets of gastric biopsies. Aliment Pharmacol Ther.

[55] Bornschein J, Tran-Nguyen T, Fernandez-Esparrach G, Ash S, Balaguer F, Bird-Lieberman EL (2021). Biopsy sampling in upper gastrointestinal endoscopy: a survey from 10 tertiary referral centres across Europe. Dig Dis.

[56] Buxbaum JL, Hormozdi D, Dinis-Ribeiro M, Lane C, Dias-Silva D, Sahakian A (2017). Narrow-band imaging versus white light versus mapping biopsy for gastric intestinal metaplasia: a prospective blinded trial. Gastrointest Endosc.

[57] Faknak N, Pittayanon R, Tiankanon K, Lerttanatum N, Sanpavat A, Klaikaew N (2022). Performance status of targeted biopsy alone versus Sydney protocol by non-NBI expert gastroenterologist in gastric intestinal metaplasia diagnosis. Endosc Int Open.

[58] Desai M, Boregowda U, Srinivasan S, Kohli DR, Al Awadhi S, Murino A (2021). Narrow band imaging for detection of gastric intestinal metaplasia and dysplasia: a systematic review and meta-analysis. J Gastroenterol Hepatol.

[59] Chiang TH, Chang WJ, Chen SLS, Yen AMF, Fann JCY, Chiu SYH (2021). Mass eradication of Helicobacter pylori to reduce gastric cancer incidence and mortality: a long-term cohort study on Matsu Islands. Gut.

[60] Sobrino-Cossío S, Teramoto-Matsubara O, Emura F, Araya R, Arantes V, Galvis-García ES (2022). Usefulness of optical enhancement endoscopy combined with magnification to improve detection of intestinal metaplasia in the stomach. Endosc Int Open.

[61] Wu CCH, Namasivayam V, Li JW, Khor CJL, Fock KM, Law NM (2021). A prospective randomized tandem gastroscopy pilot study of linked color imaging versus white light imaging for detection of upper gastrointestinal lesions. J Gastroenterol Hepatol.

[62] Yao K, Anagnostopoulos GK, Ragunath K (2009). Magnifying endoscopy for diagnosing and delineating early gastric cancer. Endoscopy.

[63] Yoshifuku Y, Sanomura Y, Oka S, Kuroki K, Kurihara M, Mizumoto T (2017). Clinical usefulness of the VS classification system using magnifying endoscopy with Blue laser imaging for early gastric cancer. Gastroenterol Res Pract.

[64] Pimentel-Nunes P, Libânio D, Lage J, Abrantes D, Coimbra M, Esposito G (2016). A multicenter prospective study of the real-time use of narrow-band imaging in the diagnosis of premalignant gastric conditions and lesions. Endoscopy.

[65] Di L, Wu H, Zhu R, Li Y, Wu X, Xie R (2017). Multi-disciplinary team for early gastric cancer diagnosis improves the detection rate of early gastric cancer. BMC Gastroenterol.

[66] Manfredi G, Pedaci M, Iiritano E, Alicante S, Romeo S (2023). Impact of improved upper endoscopy quality on detection of gastric precancerous lesions. Eur J Gastroenterol Hepatol.

[67] Yao K, Uedo N, Muto M, Ishikawa H, Cardona HJ, Filho ECC (2016). Development of an E-learning system for the endoscopic diagnosis of early gastric cancer: an international multicenter randomized controlled trial. EBioMedicine.

[68] Omura H, Yoshida N, Hayashi T, Miwa K, Takatori H, Tsuji H (2017). Interobserver agreement in detection of “white globe appearance” and the ability of educational lectures to improve the diagnosis of gastric lesions. Gastric Cancer.

[69] Januszewicz W, Witczak K, Wieszczy P, Socha M, Turkot MH, Wojciechowska U (2022). Prevalence and risk factors of upper gastrointestinal cancers missed during endoscopy: a nationwide registry-based study. Endoscopy.

[70] Pimenta-Melo AR, Monteiro-Soares M, Libânio D, Dinis-Ribeiro M (2016). Missing rate for gastric cancer during upper gastrointestinal endoscopy: a systematic review and meta-Analysis. Eur J Gastroenterol Hepatol.

[71] Wu L, Shang R, Sharma P, Zhou W, Liu J, Yao L (2021). Effect of a deep learning-based system on the miss rate of gastric neoplasms during upper gastrointestinal endoscopy: a single-centre, tandem, randomised controlled trial. Lancet Gastroenterol Hepatol.

[72] Arribas J, Antonelli G, Frazzoni L, Fuccio L, Ebigbo A, Van Der Sommen F (2021). Standalone performance of artificial intelligence for upper GI neoplasia: a meta-analysis. Gut.

[73] Luo D, Kuang F, Zhou M, Liu X, Luo X (2022). Artificial intelligence-assisted endoscopic diagnosis of early upper gastrointestinal cancer: a systematic review and meta-analysis. Front Oncol.

[74] Libanio D, Antonelli G, Marijnissen F, Spaander MCW, Hassan C, Dinis-Ribeiro M (2024). Combined gastric and colorectal cancer endoscopic screening may be cost-effective in Europe with the implementation of artificial intelligence: an economic evaluation. Eur J Gastroenterol Hepatol.

[75] de Vries AC, van Grieken NCT, Looman CWN, Casparie MK, de Vries E, Meijer GA (2008). Gastric cancer risk in patients with premalignant gastric lesions: a nationwide cohort study in The Netherlands. Gastroenterology.

[76] Price AB (1991). The Sydney system: histological division. J Gastroenterol Hepatol.

[77] Crafa P, Russo M, Miraglia C, Barchi A, Moccia F, Nouvenne A (2018). From Sidney to OLGA: an overview of atrophic gastritis. Acta Biomed.

[78] Capelle LG, de Vries AC, Haringsma J, Borg Ter, de Vries RA, Bruno MJ (2010). The staging of gastritis with the OLGA system by using intestinal metaplasia as an accurate alternative for atrophic gastritis. Gastrointest Endosc.

[79] Rugge M, DE Boni M, Pennelli G, DE Bona M, Giacomelli L, Fassan M (2010). Gastritis OLGA-staging and gastric cancer risk: a twelve-year clinico-pathological follow-up study. Aliment Pharmacol Ther.

[80] Pimentel-Nunes P, Libânio D, Marcos-Pinto R, Areia M, Leja M, Esposito G (2019). Management of epithelial precancerous conditions and lesions in the stomach (MAPS II): European Society of Gastrointestinal Endoscopy (ESGE), European Helicobacter and Microbiota Study Group (EHMSG), European Society of Pathology (ESP), and Sociedade Portuguesa de Endoscopia Digestiva (SPED) guideline update 2019. Endoscopy.

[81] Piazuelo MB, Bravo LE, Mera RM, Camargo MC, Bravo JC, Delgado AG (2021). The Colombian chemoprevention trial: 20-year follow-up of a cohort of patients with gastric precancerous lesions. Gastroenterology.

[82] Rugge M, Fassan M, Pizzi M, Pennelli G, Nitti D, Farinati F (2011). Operative Link for Gastritis Assessment gastritis staging incorporates intestinal metaplasia subtyping. Hum Pathol.

[83] Rugge M, Genta RM, Fassan M, Valentini E, Coati I, Guzzinati S (2018). OLGA gastritis staging for the prediction of gastric cancer risk: a long-term follow-up study of 7436 patients. Am J Gastroenterol.

[84] Fang S, Fu Y, Wang L, Qing X, Luo X (2022). The role of the endoscopic grading of gastric intestinal metaplasia in assessing gastric cancer risk: a systematic review and meta-analysis. Front Oncol.

[85] Yue H, Shan L, Bin L (2018). The significance of OLGA and OLGIM staging systems in the risk assessment of gastric cancer: a systematic review and meta-analysis. Gastric Cancer.

[86] Kimura K, Takemoto T (1969). An endoscopic recognition of the atrophic border and its significance in chronic gastritis. Endoscopy.

[87] Wei N, Zhou M, Lei S, Yang L, Duan Z, Zhang Y (2022). From part to whole, operative link on to endoscopic grading of gastric intestinal metaplasia, pathology to endoscopy: gastric intestinal metaplasia graded by endoscopy. Future Oncol.

[88] Pimentel-Nunes P, Dinis-Ribeiro M, Soares J, Marcos-Pinto R, Santos C, Rolanda C (2012). A multicenter validation of an endoscopic classification with narrow band imaging for gastric precancerous and cancerous lesions. Endoscopy.

[89] Esposito G, Pimentel-Nunes P, Angeletti S, Castro R, Libânio D, Galli G (2019). Endoscopic grading of gastric intestinal metaplasia (EGGIM): a multicenter validation study. Endoscopy.

[90] Fang S, Fu Y, Wang L, Qing X, Luo X (2022). The role of the endoscopic grading of gastric intestinal metaplasia in assessing gastric cancer risk: a systematic review and meta-analysis. Front Oncol.

[91] Cubiella J, Pérez Aisa Á, Cuatrecasas M, Díez Redondo P, Fernández Esparrach G, Marín-Gabriel JC (2021). Documento de posicionamiento de la AEG, la SEED y la SEAP sobre cribado de cáncer gástrico en poblaciones con baja incidencia. Gastroenterol Hepatol.

[92] Gupta S, Li D, El Serag HB, Davitkov P, Altayar O, Sultan S (2020). AGA clinical practice guidelines on management of gastric intestinal metaplasia. Gastroenterology.

[93] Lahner E, Zagari RM, Zullo A, Di Sabatino A, Meggio A, Cesaro P (2019). Chronic atrophic gastritis: natural history, diagnosis and therapeutic management. A position paper by the Italian society of hospital gastroenterologists and digestive endoscopists [aigo], the Italian society of digestive endoscopy [SIED], the Italian Society of Gastroenterology [SIGE], and the Italian society of internal Medicine [SIMI]. Dig Liver Dis.

[94] Fischbach W, Bornschein J, Hoffmann JC, Koletzko S, Link A, Macke L (2024). Update S2k-guideline Helicobacter pylori and gastroduodenal ulcer disease of the German society of Gastroenterology, digestive and metabolic diseases (DGVS). Z Gastroenterol.

[95] Banks M, Graham D, Jansen M, Gotoda T, Coda S, di Pietro M (2019). British Society of Gastroenterology guidelines on the diagnosis and management of patients at risk of gastric adenocarcinoma. Gut.

[96] Rollán A, Cortés P, Calvo A, Araya R, Bufadel ME, González R (2014). Diagnóstico precoz de cáncer gástrico: Propuesta de detección y seguimiento de lesiones premalignas gástricas: protocolo ACHED. Rev Med Chil.

[97] Rodríguez-de-Santiago E, Frazzoni L, Fuccio L, van Hooft JE, Ponchon T, Hassan C (2020). Digestive findings that do not require endoscopic surveillance – reducing the burden of care: European Society of Gastrointestinal Endoscopy (ESGE) Position Statement. Endoscopy.

[98] Lahner E, Hassan C, Esposito G, Carabotti M, Zullo A, Dinis-Ribeiro M (2017). Cost of detecting gastric neoplasia by surveillance endoscopy in atrophic gastritis in Italy: a low risk country. Dig Liver Dis.

[99] Lenti MV, Miceli E, Lahner E, Natalello G, Massironi S, Schiepatti A (2024). Distinguishing features of autoimmune gastritis depending on previous H. pylori infection or positivity to anti-parietal cell antibodies: results from the Autoimmune gastRitis Italian netwOrk Study grOup (ARIOSO). Am J Gastroenterol.

[100] Yeh JM, Hur C, Kuntz KM, Ezzati M, Goldie SJ (2010). Cost-effectiveness of treatment and endoscopic surveillance of precancerous lesions to prevent gastric cancer. Cancer.

[101] Lansdorp-Vogelaar I, Meester RGS, Laszkowska M, Escudero FA, Ward ZJ, Yeh JM (2021). Cost-effectiveness of prevention and early detection of gastric cancer in Western countries. Best Pract Res Clin Gastroenterol.

[102] Areia M, Carvalho R, Cadime AT, Gonçalves F, Dinis-Ribeiro M (2013). Screening for gastric cancer and surveillance of premalignant lesions: a systematic review of cost-effectiveness studies. Helicobacter.

[103] Chinese Society of Gastroenterology CCG of CS of GCMA (2023). Guidelines for diagnosis and treatment of chronic gastritis in <scp>China</scp> (2022, <scp>Shanghai</scp>. J Dig Dis.

